# Double Strand Break DNA Repair occurs via Non-Homologous End-Joining in Mouse MII Oocytes

**DOI:** 10.1038/s41598-018-27892-2

**Published:** 2018-06-26

**Authors:** Jacinta H. Martin, Elizabeth G. Bromfield, R. John Aitken, Tessa Lord, Brett Nixon

**Affiliations:** 1 Priority Research Centre for Reproductive Science, School of Environmental and Life Sciences, The University of Newcastle, Callaghan, NSW 2308 Australia; 2 Preganancy and Reproduction Program, Hunter Medical Research Institute, New Lambton Heights, NSW 2305 Australia; 3 School of Molecular Biosciences, Centre for Reproductive Biology, College of Veterinary Medicine, Washington State University, Pullman, WA 99164 USA

## Abstract

The unique biology of the oocyte means that accepted paradigms for DNA repair and protection are not of direct relevance to the female gamete. Instead, preservation of the integrity of the maternal genome depends on endogenous protein stores and/or mRNA transcripts accumulated during oogenesis. The aim of this study was to determine whether mature (MII) oocytes have the capacity to detect DNA damage and subsequently mount effective repair. For this purpose, DNA double strand breaks (DSB) were elicited using the topoisomerase II inhibitor, etoposide (ETP). ETP challenge led to a rapid and significant increase in DSB (P = 0.0002) and the consequential incidence of metaphase plate abnormalities (P = 0.0031). Despite this, ETP-treated MII oocytes retained their ability to participate in *in vitro* fertilisation, though displayed reduced developmental competence beyond the 2-cell stage (P = 0.02). To account for these findings, we analysed the efficacy of DSB resolution, revealing a significant reduction in DSB lesions 4 h post-ETP treatment. Notably, this response was completely abrogated by pharmacological inhibition of key elements (DNA-PKcs and DNA ligase IV) of the canonical non-homologous end joining DNA repair pathway, thus providing the first evidence implicating this reparative cascade in the protection of the maternal genome.

## Introduction

All cells within the human body encounter potentially thousands of DNA lesions on a daily basis owing to their exposure to a variety of internal and environmental factors^1^. If left unresolved, these lesions can potentially lead to mutations and broader-scale genomic aberrations that compromise cell viability and/or elevate predisposition to diseases such as cancer^2,3^. To ameliorate this threat, most cells are capable of mounting stringent DNA damage responses, which incorporate pathways for surveillance of DNA damage and mediation of its immediate repair. In somatic cells, molecular pathways of DNA repair include non-homologous end joining (NHEJ), homologous recombination (HR), mismatch repair (MMR) and nucleotide excision repair (NER)^4^. These pathways are generally very well-characterised, with a defined subset of the proteome devoted to sequentially detecting DNA lesions, remodelling chromatin and effecting DNA repair. In contrast to somatic cells however, there is less known about the DNA damage response in the oocyte beyond the ongoing maintenance of the resting population of primordial follicles by the Tap63 pathway^5–8^. Moreover, unlike somatic cells that can dynamically upregulate the synthesis of DNA damage response machinery following genotoxic insult^9,10^, the mature ovulatory stage oocyte is transcriptionally silent and thus incapable of mounting an equivalent response. Instead, these cells must depend on endogenous stores of pre-synthesised proteins and/or mRNA transcripts accumulated during oogenesis to enact DNA repair^11^.

The balance of evidence suggests that, beyond the follicular phases of development, immature oocytes and those arrested in the germinal vesical stage (GV), contain relatively inefficient DNA damage response mechanisms and are therefore largely refractory to DNA repair^12,13^. For instance, despite harbouring sufficient DNA damage to delay germinal vesicle breakdown (GVBD), reduce polar body extrusion rates and alter spindle assembly checkpoint dynamics, GV stage oocytes nonetheless retain the ability to progress to the fertilisation competent MII stage of development^14–16^. The absence of efficient DNA damage responses capable of effecting repair, or stalling development, in GV oocytes carries with it the attendant risk of propagating DNA lesions into the embryo. This situation appears to arise, at least in part, from an inability of the GV oocytes to activate the master regulator of the DNA damage response pathway, ataxia telangiectasia mutated kinase (ATM) unless the levels of DNA damage surpass a particularly high threshold^14^. In the presence of such severe damage, it has been shown that the GV oocyte will arrest due to activation of an alternative, oocyte-specific checkpoint known as the spindle assembly checkpoint (SAC), which prevents exit from M-phase^12,13^. Although still relatively insensitive, the SAC may act to protect against both aneuploidy and the inheritance of DNA damage by preventing the production of abnormal mature oocytes and subsequent embryos^12^. While it is not yet clear why immature oocytes fail to activate major DNA damage response or repair factors, it has yet to be determined whether mature MII phase oocytes are also recalcitrant to DNA repair, or whether this later developmental stage is equipped to resolve DNA lesions and thus mitigate the possibility of DNA damaged oocytes participating in fertilisation^17^.

The impetus to study this mature stage stems from our recent findings that metaphase II (MII) oocytes are inherently susceptible to DSB DNA damage, owing to an inability to engage membrane defence proteins, such as those of the permeability glycoprotein family, which would otherwise counter the influx of genotoxic agents into the ooplasm^18,19^. Moreover, comparative proteomic analyses of mouse MII oocytes performed in an independent study have identified a total of 53 proteins putatively involved in the DNA damage response and DNA repair related processes, including: NER, single strand break repair (SSB), double strand break repair (DSB) and base excision repair (BER)^20^. Notably, 35 (66%) of these proteins were upregulated in the MII oocyte when compared to their GV oocyte and zygote counterparts^20^. This previously unappreciated level of enrichment of DNA damage response proteins may reflect their integral role in ensuring the integrity of the maternal genome required for the correct initiation of embryonic transcription. The current study was undertaken to expand our understanding of the extant molecular repair mechanisms in the post-ovulatory oocyte and the cell’s capacity for the repair of DSB DNA damage. Such mechanistic understanding is of critical importance in protecting the genetic integrity of the oocyte to allow for its unimpeded transition through embryogenesis.

## Results

### Exposure to chemotherapeutic agents induces DNA damage and metaphase plate misalignment in MII oocytes

**Figure 1:**
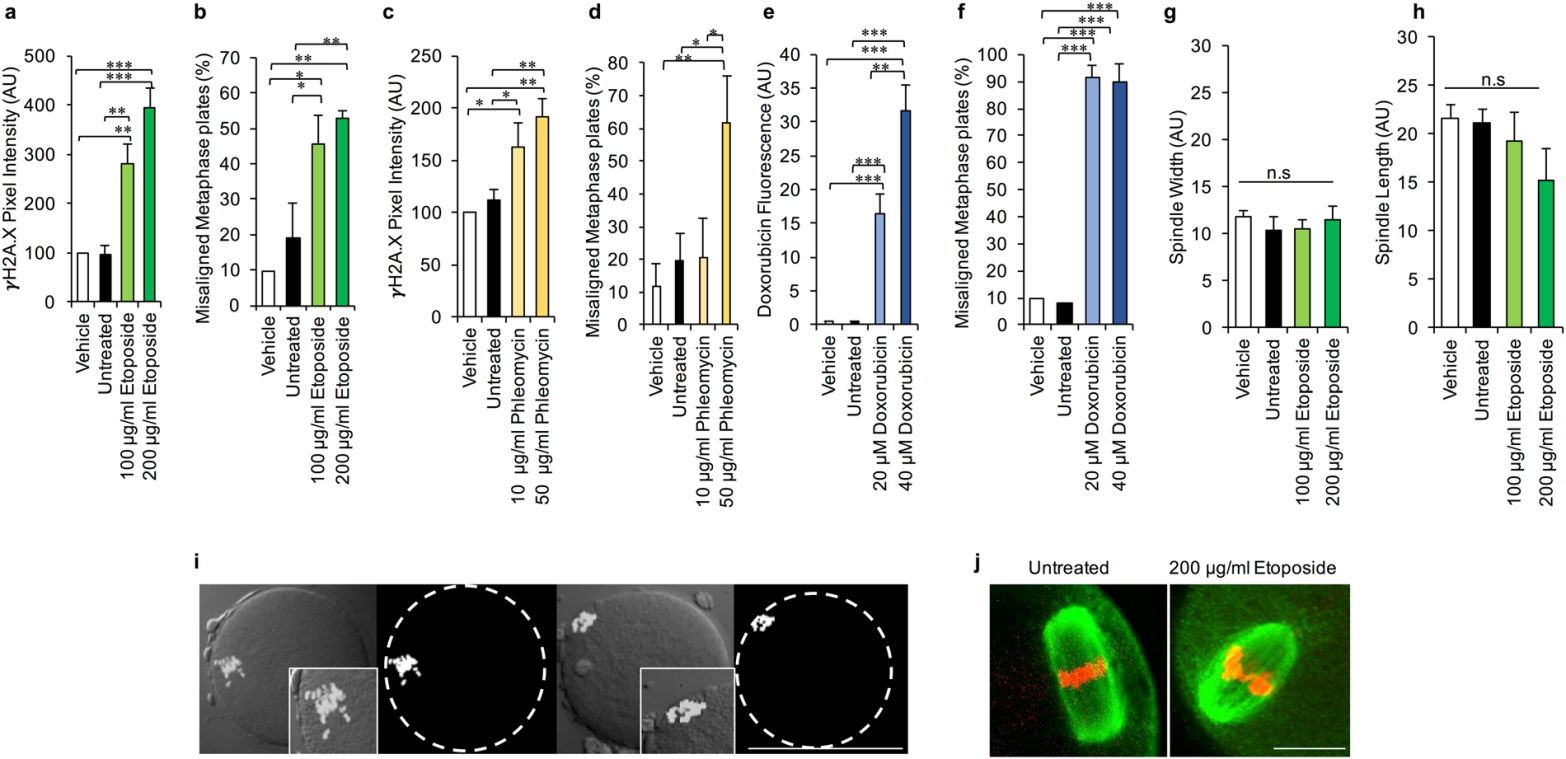
Assessment of the vulnerability of the mouse ovulatory stage oocyte to genotoxic stressors. (**a,c,e**) MII oocytes demonstrated a dose-dependent increase in γH2A.X and doxorubicin labelling (DOX treatment) at the metaphase plate following exposure to ETP, PHL or DOX. (**b,d,f**) γH2A.X labelling was accompanied by a pronounced, concomitant increase in metaphase plate abnormalities. (**g,h**) In addition spindle width and length were analysed as an early predictor of embryonic competency. This analysis revealed that ETP-induced perturbation of the metaphase plate was not associated with overt alterations in either measurement. (**i–j**) Representative images illustrate precocious chromatin segregation in MII oocytes as well as (**j**) confirm the retention of normal spindle width and length (green = tubulin, red = pseudocoloured DAPI). Statistical significance was determined using ANOVA, Tukey–Kramer HSD and Student’s t-tests. *p ≤ 0.05, **p ≤ 0.01 and ***p ≤ 0.001. Each experiment was conducted on a minimum of three biological replicates, with each replicate comprising a minimum of 30 oocytes. All data are expressed as means ± S.E.M. Scale bars in (**i**) and (**j**) =80 μm and  10 μm, respectively.

To assess the vulnerability of the ovulatory stage oocyte to genotoxic stressors, mouse MII oocytes were liberated from the ampulla and cultured in the presence of the chemotherapeutic agents etoposide (ETP), phleomycin (PHL) or doxorubicin (DOX), each of which are capable of inducing potent DSB DNA damage. Oocytes were subsequently assessed for the presence of DNA DSBs via measurement of the relative intensity of either γH2A.X labelling (ETP and PHL treatments) or DOX fluorescence within the vicinity of the metaphase plate (DOX treatment; DOX has a fluorescent signature that correlates with DNA damage^21^) (Fig. 1). As anticipated, neither untreated nor vehicle (DMSO) control oocytes presented with any appreciable evidence of DSB DNA damage. However, consistent with previous studies^12,13,19^ significant, dose-dependent increases in criteria indicative of DSB DNA damage were recorded following administration of either ETP (Fig. 1a, P = 0.0002), PHL (Fig. 1c, P = 0.0096) or DOX (Fig. 1e, P < 0.0001). The pervasive impact of such treatments also manifested in the form of marked increases in the incidence of metaphase plate alignment abnormalities (Fig. 1i; ETP P = 0.0031, PHL P = 0.0264 and DOX P < 0.0001) and, in the case of ETP and PHL this response was again dose-dependent (Fig. 1b,d). Indeed, approximately half of all oocytes exposed to the highest concentrations of ETP (200 µg/ml; P = 0.0062) or PHL (50 µg/ml; P = 0.0171) experienced pronounced misalignment of their metaphase plates (Fig. 1b,d). This effect was even more pronounced in the case of DOX treatment, with ~90% of exposed oocytes responding with misalignment of their metaphase plates at the lowest dose tested (Fig. 1f; P < 0.0001).

### Fertilising potential of etoposide treated MII oocytes

On the basis of the consistent response to each of the three chemotherapeutic agents used (Fig. 1), herein the decision was made to focus on the single insult of ETP treatment in order to begin to investigate: (i) the physiological consequences of this genotoxic agent on the developmental competence of MII oocytes and (ii) whether the MII oocyte is capable of engaging in DNA repair. As an initial line of enquiry, we sought to determine if ETP-induced misalignment of the metaphase plate was associated with additional perturbation of meiotic spindle characteristics. Specifically, we focused our assessment on meiotic spindle length and width (Fig. S1), owing to the utility of spindle morphology as a predictor of embryonic developmental potential^22–27^. Perhaps surprisingly, this analysis revealed that ETP-induced perturbation of the metaphase plate was not accompanied by appreciable alterations in either spindle length (Fig. 1g,j) or spindle width (Fig. 1h,j), thus raising the prospect that ETP treated MII oocytes may retain some developmental capacity. Accordingly, this possibility was directly assessed via *in vitro* fertilisation (IVF) and early embryonic development assays.

**Figure 2:**
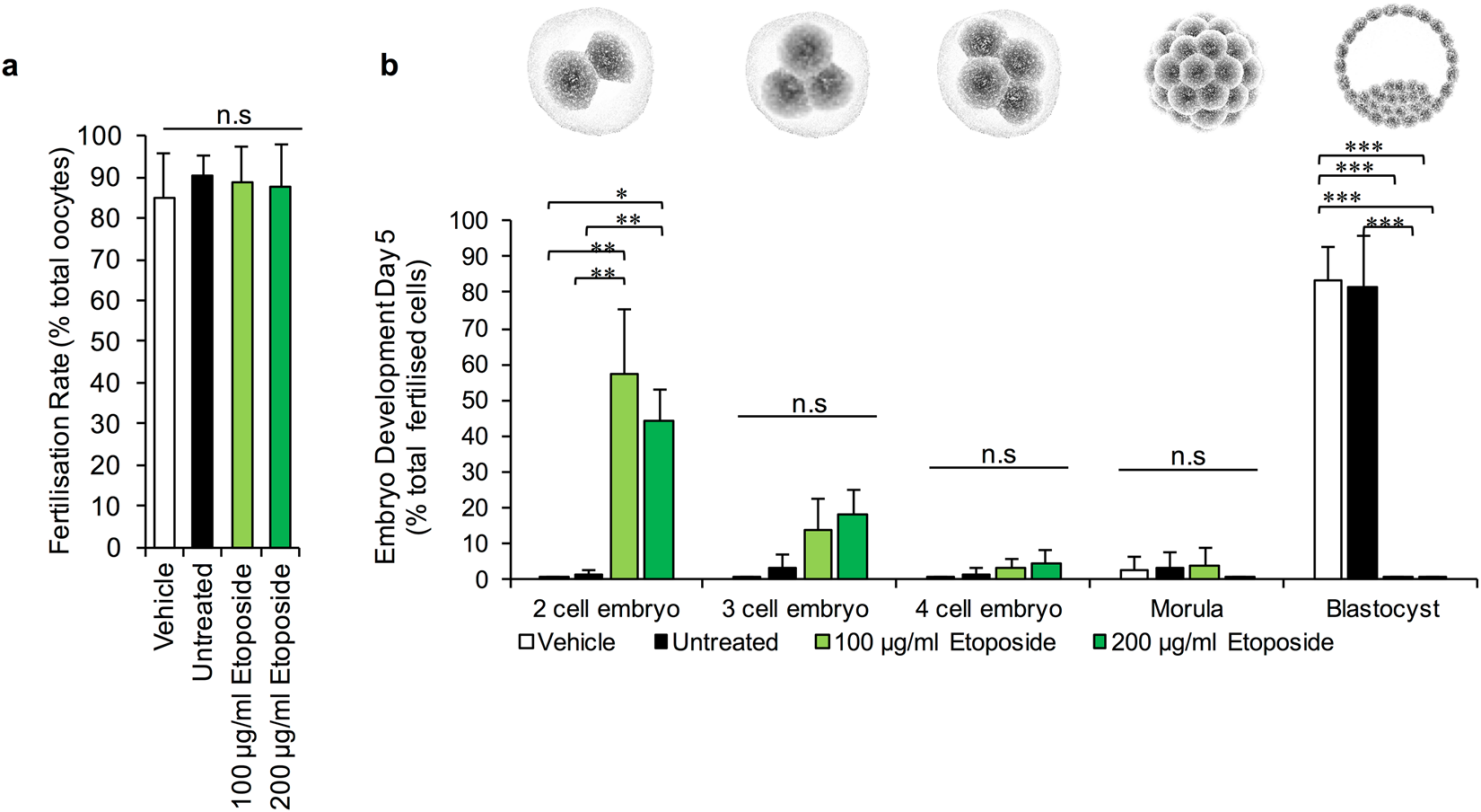
Physiological consequences of etoposide exposure. The developmental competence of ETP exposed MII oocytes was directly assessed via *in vitro* fertilisation and early embryonic development assays. For this purpose oocytes were co-cultured with approximately 2 × 10^5^ capacitated spermatozoa for 4 h at 37 °C. (**a**) ETP-treated MII oocytes retained their competence to participate in fertilisation. (**b**) However, ETP-treated MII oocytes were not able to support embryonic development through to blastocyst formation (i.e. 5 days post-fertilisation) whereby 40–60% of embryos failed to progress beyond the two cell stage of development. Statistical significance was determined using ANOVA, and Tukey–Kramer HSD. *p ≤ 0.05, **p ≤ 0.01 and ***p ≤ 0.001. Each experiment was conducted on a minimum of three biological replicates, with the total number of embryos assessed across these replicates being: vehicle = 95; untreated = 93; 100 µg/ml  ETP = 95; 200 µg/ml ETP = 86. All data are expressed as means ± S.E.M. Scale bar = 80 μm.

IVF assays revealed that ETP-treated MII oocytes retained their competence to participate in fertilisation, with fertilisation rates of >80% documented among populations of ETP-exposed oocytes; rates that proved indistinguishable from those recorded in unexposed or vehicle control oocyte groups (Fig. 2a). We also confirmed that the total number of pronuclei remained unchanged (i.e. 1 of maternal and 1 of paternal origin) in zygotes resulting from fertilisation of ETP-treated oocytes. While such results are consistent with the lack of overt defects in the morphometric characteristics of the meiotic spindle (Fig. 1), they are nonetheless at odds with the elevated levels of DNA damage these treated oocytes harbour (Fig. 1) and the fact that they present with significant misalignment of the metaphase plate. Consequently, our analysis was extended to determine the competency of ETP-treated MII oocytes to support normal embryonic development through to blastocyst formation (i.e. up to 5 days post-fertilisation) (Fig. 2b).

As illustrated in Fig. 2b, as many as 40–60% of embryos arising from ETP-treated MII oocytes failed to progress beyond the two-cell stage of development (100 μg/ml ETP, *P* = 0.004 and 200 μg/ml ETP, *P* = 0.02, compared to the controls). A modest number of the remaining embryos (~13–18%) progressed to the three - four cell stage, whilst only a very small subset (3%) developed beyond this point (Fig. 2b). This latter cohort were all arrested at the morula stage prior to proceeding to blastocyst formation. In stark contrast, >80% of embryos conceived from untreated or vehicle control oocytes experienced normal development through to the hatching and expanding blastocyst stages (Fig. 2d) (P = 0.0001). Based on these collective data we infer that ETP can dramatically attenuate both the genomic integrity and developmental competency of the mouse MII oocyte and the embryos that arise through the fertilisation of these cells.

### Investigation of the physiological characteristics of arrested two cell embryos

**Figure 3:**
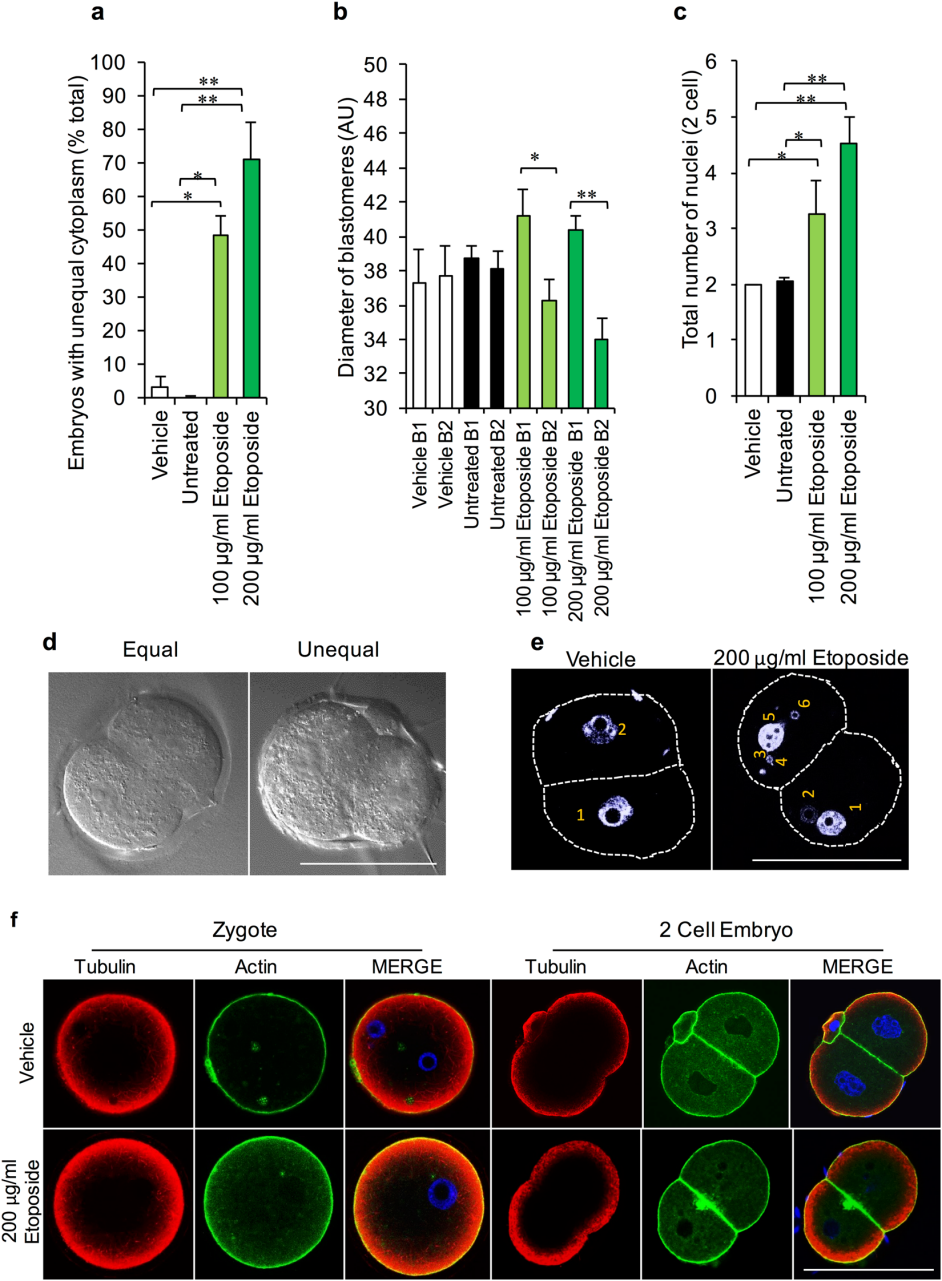
Morphometric analysis of arrested two cell embryos generated from the etoposide-treated MII oocytes. (**a–d**) Embryos borne of ETP-exposed MII oocytes were characterised by significant fragmentation and unequal cytoplasm partitioning, accompanied by an increase in diameter in the notably larger blastomeres (greater than 25% difference). (**c–e**) Accordingly, nuclear integrity was also analysed, revelaing that daughter cells derived from ETP-treated MII oocytes contained additional multinucleate structures in addition to the pronucleus. (**f**) Localisation of the actin and tubulin networks of the preimplantation zygote and 2 cell embryos were analysed for their potential contribution to the unequal cytoplasmic partitioning. However, neither embryonic stage exhibited apparent modifications in labelling intensity or distribution patterns of tubulin or actin irrespective of the treatment they received. Statistical significance was determined using ANOVA and Student’s t-tests. *p ≤ 0.05, **p ≤ 0.01 and ***p ≤ 0.001. Each experiment was conducted on a minimum of three biological replicates, with each replicate comprising a minimum of 30 oocytes. All data are expressed as means ± S.E.M. Scale bar = 80 μm.

To better define the characteristics of ETP-induced restriction of blastomere proliferative capacity, two cell embryos generated from the ETP-treated population of MII oocytes were subjected to morphometric analysis with a particular focus on cytoplasmic partitioning and nuclear integrity. This analysis revealed that embryos originating from ETP-exposed MII oocytes presented with significantly elevated fragmentation and unequal cytoplasm partitioning (*P* = 0.0034) (Fig. 3a). The latter commonly manifested in the form of one ‘large’ blastomere accompanied by a significantly ‘smaller’ counterpart (Fig. 3b; denoted as B1 and B2, respectively). Notably, the abnormally large blastomeres were characterised by a significant disparity in diameter (*P* = 0.0495 and *P* = 0.016, respectively) when compared to the blastomeres present within control embryos (Fig. 3b,d). Such findings raise the prospect that the cytoskeletal network responsible for faithful completion of blastomere cytokinesis is compromised by the legacy of oocyte ETP exposure. This interpretation is in keeping with reports that senescence-like phenotypes induced by cytostatic drugs such as ETP (and DOX) are coupled to alterations in the integrity of cytoskeletal elements present in the treated cells^28,29^.

Accordingly, we sought to localise both the actin and tubulin networks of the preimplantation zygote and 2 cell embryos derived from these untreated and ETP-treated populations of oocytes. At the resoltuion afforded by confocal microscopy, neither embryonic stage exhibited overt changes in actin or tubulin labelling intensity or distribution patterns irrespective of the treatment they received (Fig. 3f). Notably, in contrast to the unaltered distribution of actin and tubulin in the embryos following ETP exposure, multinucleate structures were exclusively detected in the daughter cells derived from ETP-treated MII oocytes, and were not present in their control counterparts (Fig. 3c,e) (*P* = 0.005).

### DNA repair occurs via non-homologous end-joining (NHEJ)

**Figure 4:**
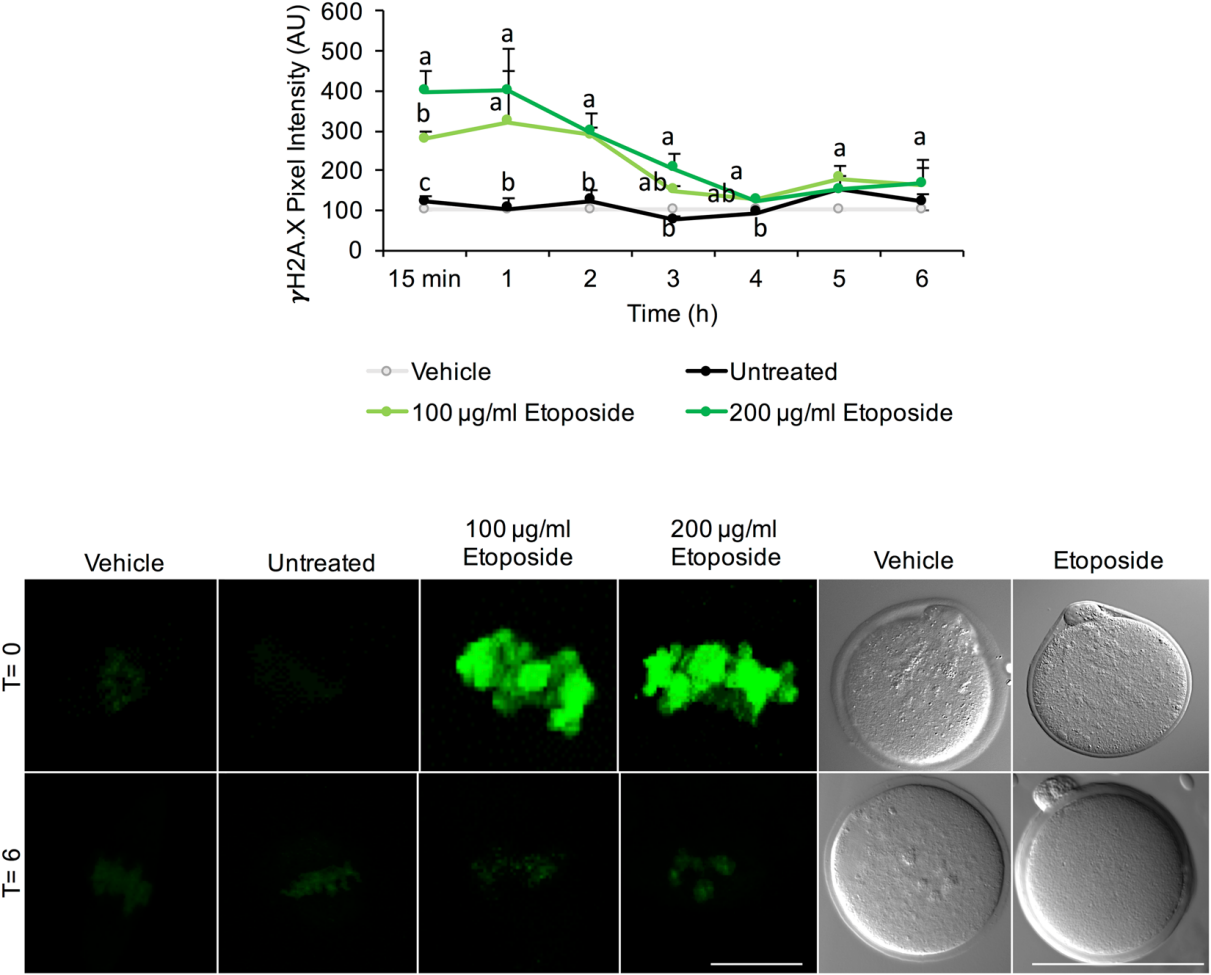
DNA repair in the MII oocyte occurs via non-homologous end-joining (NHEJ). To determine if the MII oocyte was capable of mounting DNA damage repair, γH2A.X fluorescent labelling was utilised to produce a kinetic profile of DNA repair over a recovery period of 6 h. γH2A.X labelling was significantly reduced by 1 h post-treatment; reaching basal levels by 4 h. Representative images illustrate that at 4 h post treatment, γH2A.X fluorescence intensity was comparable to the basal levels documented in untreated control oocytes. Statistical significance was determined using ANOVA and Tukey–Kramer HSD. A connecting letter plot was used to denote which groups differed significantly from each another. Each experiment was conducted on a minimum of three biological replicates, with each replicate comprising a minimum of 30 oocytes. All data are expressed as means ± S.E.M. Scale bar = 20 μm.

Having established the impact of acute ETP exposure in terms of attenuating the developmental competency of the MII oocyte, we next sought to address our second goal of determining whether this cell is capable of mounting any form of DNA damage repair in response to this genotoxic insult. For this purpose, MII oocytes were treated with ETP prior to monitoring the intensity of γH2A.X fluorescent labelling over a recovery period of up to 6 h^30^. Under these experimental conditions, the intensity of γH2A.X foci was significantly elevated above that of untreated control oocytes as early as 15 min after ETP administration in all oocytes (100%). Thereafter, γH2A.X labelling appeared to plateau at 1 h post-treatment, before gradually decreasing to basal levels comparable to those of the untreated controls by 4 h post-treatment (Fig. 4). No further reduction in the intensity of γH2A.X labelling was observed between 4 and 6 h post-treatment. We also noted that ETP failed to elicit any overt changes in oocyte morphology irrespective of the concentration used in this study. This remained true following the initial insult and subsequent to the recovery period (T = 6 h).

**Figure 5:**
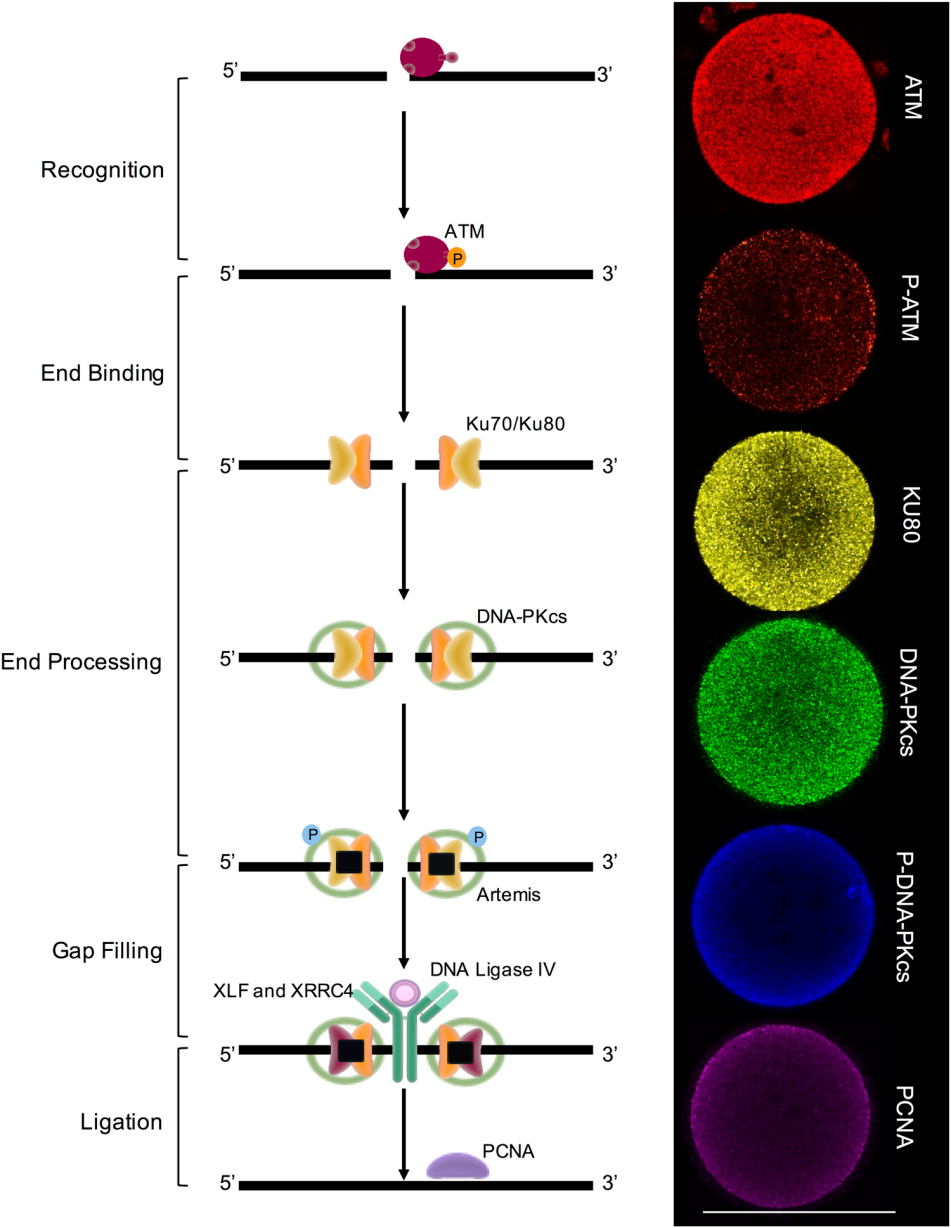
MII oocytes contain the necessary protein machinery for NHEJ. The presence and localisation of several key enzymes within the NHEJ pathway were investigated using immunocytochemistry. Ataxia telangiectasia mutated (ATM), X-ray repair complementing defective repair in Chinese hamster cells (KU80/XRCC5), DNA-dependent protein kinase catalytic subunit (DNA PKcs) and Proliferating cell nuclear antigen (PCNA) were each identified within the MII oocyte. In addition, the active phosphorylated forms of both ATM (p-Ser1981) and DNA PKcs (p-Ser2056) were also detected within these cells. In all instances, punctate fluorescence was disseminated throughout the ooplasm. Scale bar = 80 μm.

This kinetic profile of γH2A.X resolution in ETP treated oocytes is consistent with that expected following the recruitment of DSB DNA repair pathway(s) (2–6 h^31^). In this context, the repair of DSB lesions is generally mediated through two key repair pathways, homologous recombination (HR) and non-homologous end-joining (NHEJ)^4^. While we cannot specifically exclude the contribution of HR, this scenario was considered unlikely due to it’s restricted activity within the late S-G2 phases of the cell cycle^4,32^. Instead, we focused our analysis on the contribution of NHEJ in resolving the ETP induced DSB DNA damage. For this purpose, immunocytochemistry was used to investigate the presence and localisation of several enzymes that hold key functional roles within this pathway, namely: Ataxia telangiectasia mutated (ATM), X-ray repair complementing defective repair in Chinese hamster cells (KU80/XRCC5), DNA-dependent protein kinase catalytic subunit (DNA-PKcs) and Proliferating cell nuclear antigen (PCNA) (Fig. 5). In keeping with data from recent global proteomic analyses^20^, our study confirmed the expression of ATM, KU80, DNA-PKcs and PCNA in mouse MII oocytes (Fig. 5). Moreover, we were also able to detect the active, phosphorylated forms of both ATM (p-Ser1981) and DNA-PKcs (p-Ser2056)^33,34^ (Fig. 5). In all instances, the fluorescence associated with these enzymes was dispersed throughout the ooplasm and was generally characterised by punctate spatial patterning. The specificity of these labelling patterns was confirmed by the complete absence of labelling in negative controls in which the primary antibody was substituted with buffer alone. In addition to this, immunoblotting with the positive controls HEK293 and UV treated HELA lysates further confirmed the specificity of these antibodies (Fig. S2).

**Figure 6:**
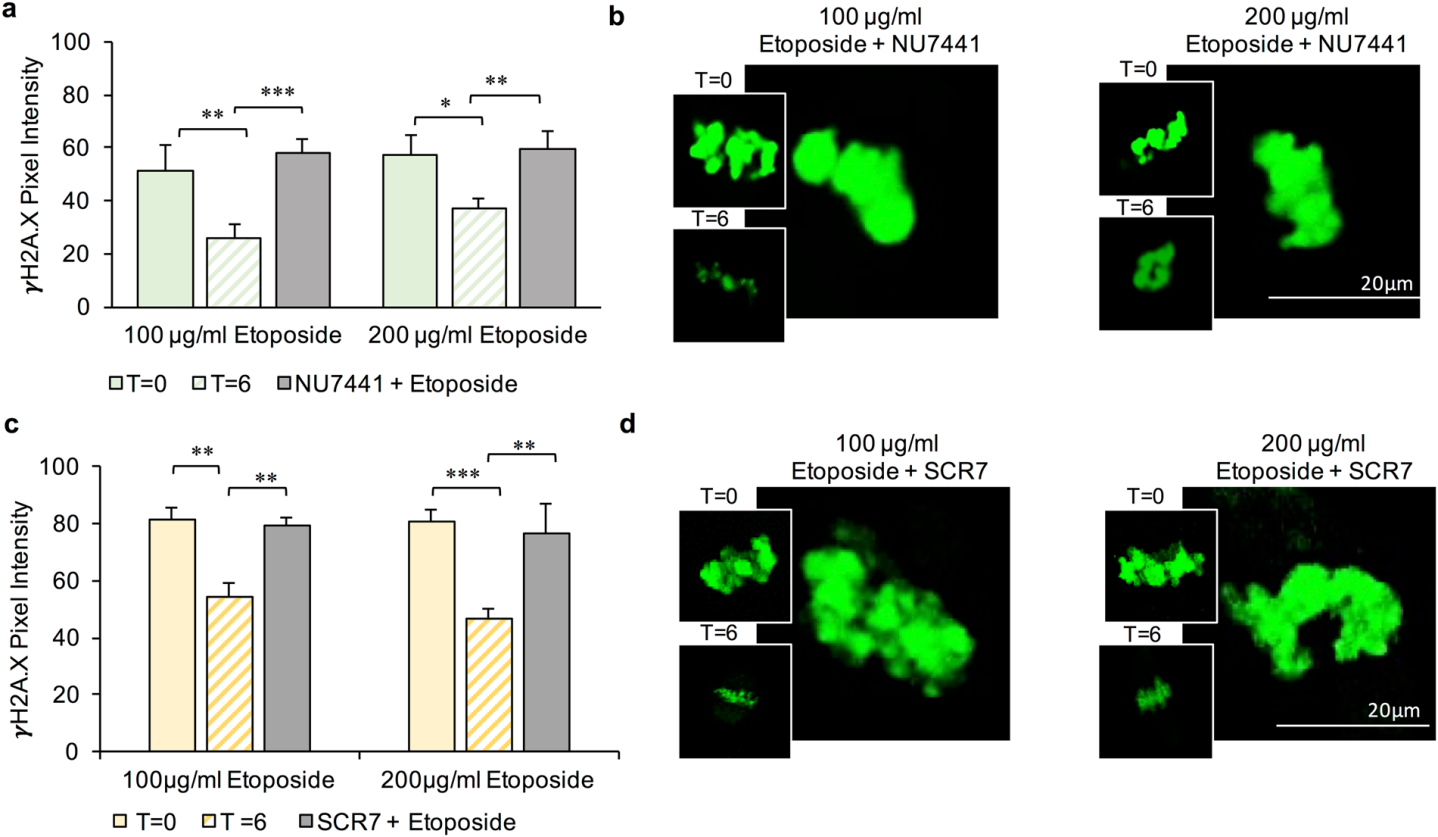
Assessment of the functional significance of NHEJ repair activity by selective pharmacological inhibition. (**a**) NU7441 (50 μM) a potent and selective antagonist of DNA-PKcs and **(c)** SCR7 (20 μM), a selective inhibitor of DNA ligase IV were incubated with ETP-treated oocytes during a recovery time course (6 h). In both instances, inhibition of DNA-PKcs or DNA ligase IV completely prohibited the resolution of γH2A.X foci. In contrast, ETP-treated oocytes not challenged with these pharmacological inhibitors experienced a significant decrease in γH2A.X labelling following an equivalent recovery period. (**b** and **d**) Representative immunofluorescence images of γH2A.X labelling of the metaphase plate are presented, with the main image corresponding to oocytes treated with (**b**) ETP + NU7441 and (**d**) ETP + SCR7. Similarly, the insets in panels (**b**) and (**d**) correspond to γH2A.X labelling of the metaphase plate in ETP treated oocytes at T = 0 (i.e. upper insets) and again at T = 6 h (lower insets). Statistical significance was determined using ANOVA and Student’s t-tests. *p ≤ 0.05, **p ≤ 0.01 and ***p ≤ 0.001. Each experiment was conducted on a minimum of three biological replicates, with each replicate comprising a minimum of 30 oocytes. All data are expressed as means ± S.E.M. Scale bar = 20  μm.

Having confirmed that MII oocytes harbour key elements of the NHEJ pathway, we next examined the functional significance of NHEJ repair activity through the application of selective pharmacological inhibitors (Fig. 6). Specifically, ETP treated oocytes were incubated in culture medium supplemented with either 50 μM NU7441 (a potent and selective agonist of DNA-PKcs)^35^ or 20 μM SCR7 (marketed as a selective inhibitor of DNA ligase IV)^36^ over a time course of 6 h (Figs 6 and S3). After this recovery period, oocytes were fixed and examined for γH2A.X fluorescent labelling as an indicator of DNA damage. This experimental strategy confirmed that inhibition of either DNA-PKcs (Fig. 6a,b; P < 0.0001) or DNA ligase IV (Fig. 6c,d; P < 0.0001) completely abrogated the resolution of γH2A.X foci. Indeed, γH2A.X labelling intensity was maintained at a level reminiscent of that achieved in all oocytes immediately following ETP treatment (NU7441: 100 μg/ml *P* = 0.3789 and 200 μg/ml *P* = 0.8407; SCR7: 100 μg/ml *P* = 0.8885, and 200 μg/ml *P* = 0.6419), even after the maximal recovery period of 6 h. Over the same time course, those ETP treated oocytes that were not challenged with pharmacological inhibitors experienced the customary decrease of γH2A.X labelling (NU7441: 100 μg/ml *P* = 0.0032 and 200 μg/ml *P* = 0.0157; SCR7: 100 μg/ml *P* = 0.0056 and 200 μg/ml *P* = 0.0005) to basal levels indistinguishable from that of untreated control cells (Fig. 6a,c and insets in Fig. 6b,d).

### NHEJ-mediated repair of ETP-induced DNA DSBs is not sufficient to rescue embryonic development

**Figure 7:**
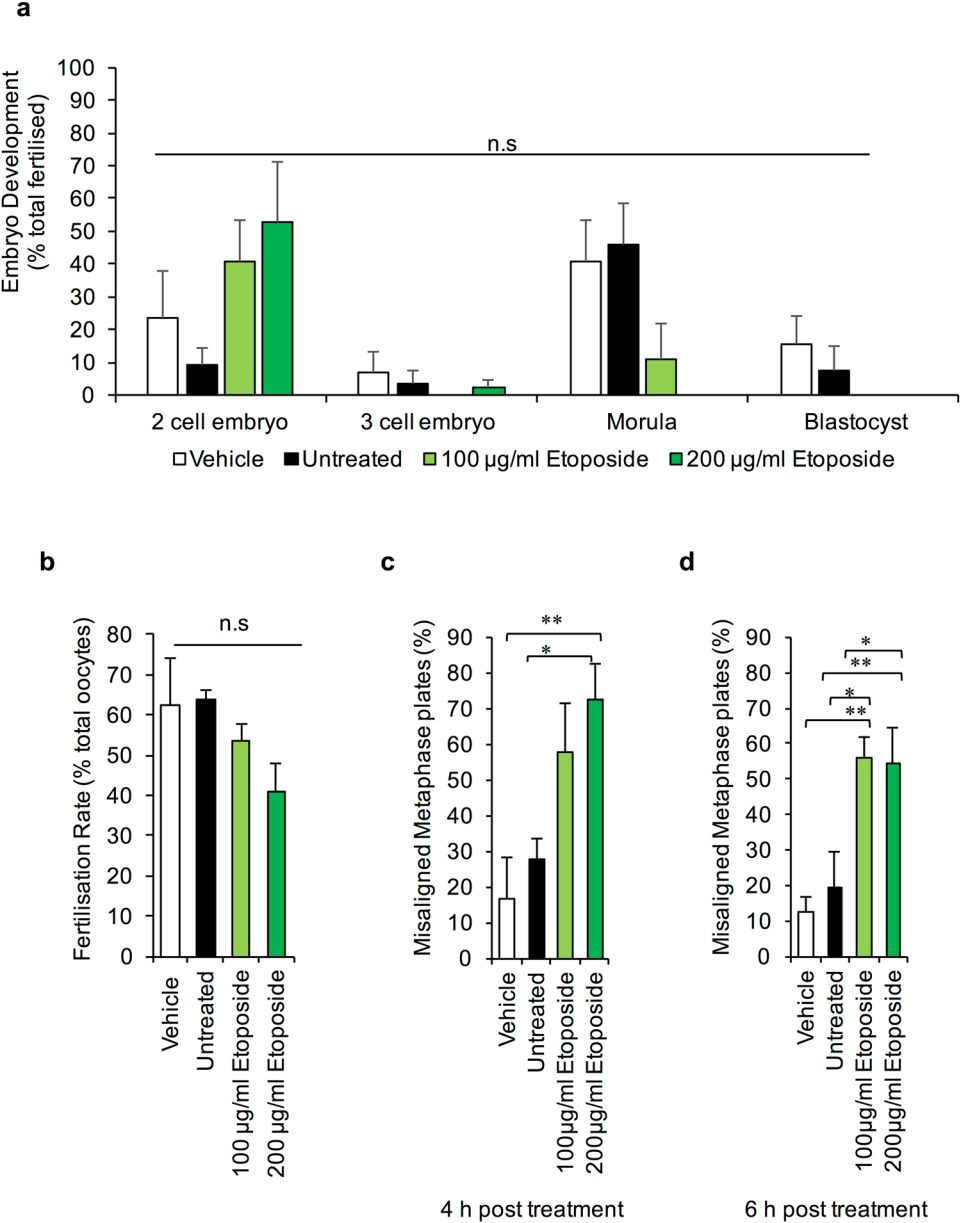
NHEJ-mediated repair of ETP-induced DNA DSBs is not sufficient to rescue embryonic development. The developmental potential of oocytes challenged with an ETP insult was analysed whereby treated oocytes were provided a minimum recovery time of 4 h prior to *in vitro* fertilisation. (**a**) Embryonic competency was not appreciably altered within the 4 h post-ETP recovery window with limited numbers of embryos progressing beyond the two-cell stage of embryogenesis. (**b**) In addition, a notable reduction in fertilisability was evident in all oocytes derived of both the treated and untreated oocytes. (**c**) Analysis of metaphase plate integrity at 4 and 6 h recovery time points indicated that neither period of post-treatment recovery was sufficient to ameliorate the incidence of metaphase plate misalignment. Statistical significance was determined using ANOVA and Tukey–Kramer HSD. *p ≤ 0.05 and **p ≤ 0.01. Each experiment was conducted on a minimum of three biological replicates, with the total number of embryos assessed across these replicates being: vehicle = 40; untreated = 50; 100 µg/ml  ETP = 40; 200 µg/ml  ETP = 27. In regard to oocytes, each experiment was conducted on a minimum of three biological replicates, with each replicate comprising a minimum of 30 oocytes. All data are expressed as means ± S.E.M.

Given that the progression of NHEJ DNA repair is generally considered to be of lower fidelity than that of HR, it was of interest to determine if this repair pathway could rescue the developmental potential of oocytes challenged with an ETP insult. Thus, ETP treated oocytes were allowed the minimum recovery time of 4 h prior to being inseminated via IVF. The embryonic competency of fertilised oocytes was subsequently assessed revealing that the extent of NHEJ taking place within 4 h post-ETP treatment was not able to appreciably alter the number of embryos progressing beyond the two-cell stage of embryogenesis (Fig. 7a). Notably, as a likely consequence of the post-ovulatory oocyte aging process^37,38^, the control (vehicle exposed and untreated) oocytes in this experiment also experienced a reduction in both their fertilisability and developmental competency (Fig. 7a,b) when compared to embryos generated from freshly isolated MII oocytes (Fig. 2). Notwithstanding this phenomenon, the deterioration in oocyte quality we encountered was certainly not of sufficient magnitude to account for the restricted proliferative capacity of blastomeres derived from ‘repaired’ oocytes. In seeking to account for these data, we assessed the integrity of the metaphase plates formed in ETP treated oocytes following 4 and 6 h recovery time points (Fig. 7c,d). Regrettably, neither period of post-treatment recovery proved sufficient to ameliorate the incidence of metaphase plate misalignment (4 h: *P* = 0.009 and 6 h: *P* = 0.0088 compared to untreated controls), with both populations characterised by alignment abnormalities equivalent to those recorded in the absence of any recovery period (Fig. 1b).

### DNA damage can be prevented by co-administration of sodium salicylate

Our collective evidence indicates that, despite the presence of an active NHEJ pathway, oocytes are unable to mount a repair pathway of sufficient efficacy to alleviate the impact of genotoxic agents such as ETP. We therefore sought to explore the utility of alternative therapeutic interventions to prevent, rather than repair, ETP damage. Specifically, we explored the potential to ameliorate the effects of ETP on the MII stage oocyte via co-administration of either N-acetylcysteine (NAC) or sodium salicylate (SS), compounds that have been proposed to modulate the effects of ETP in somatic cells^39–41^.

**Figure 8:**
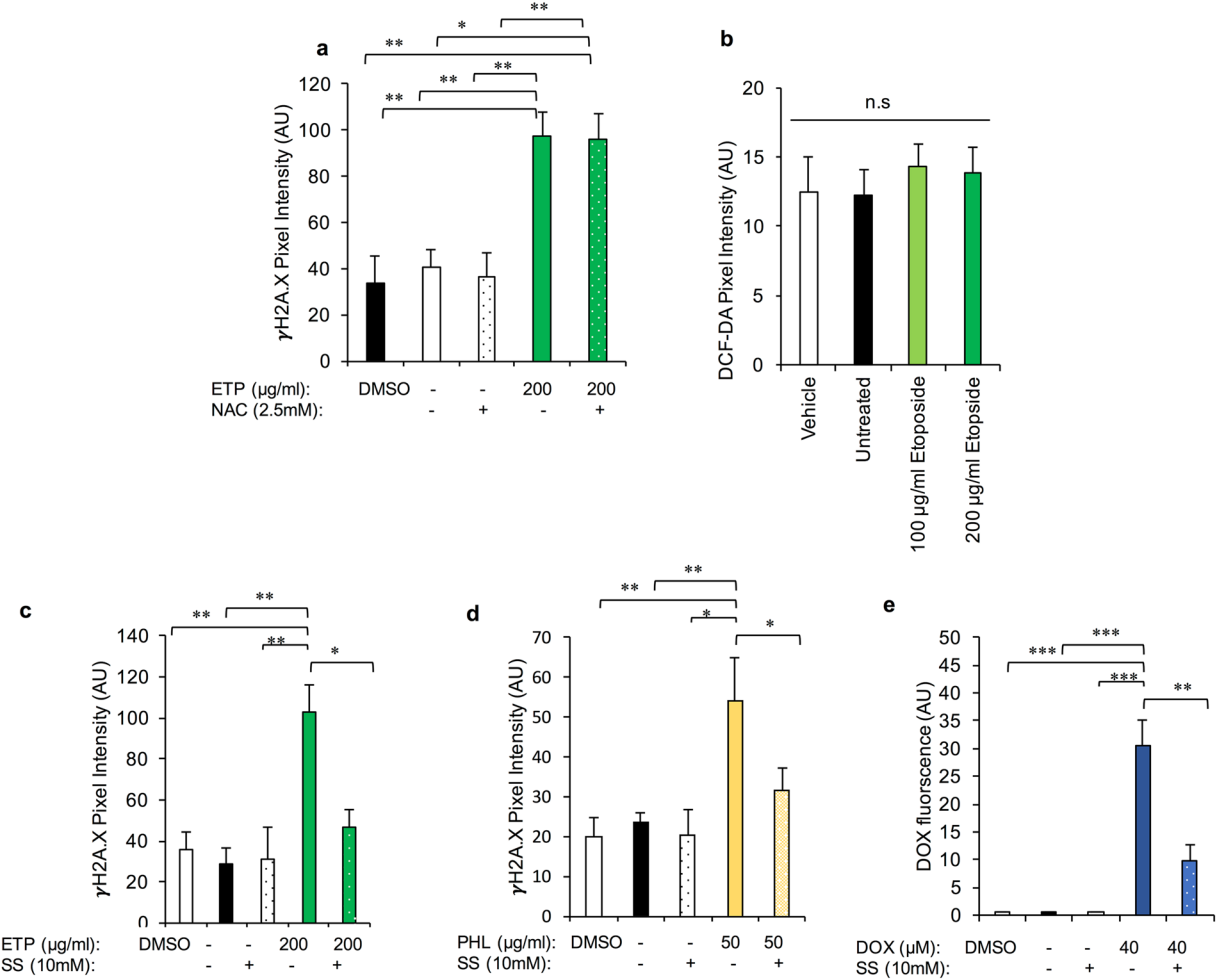
DNA damage can be prevented by co-administration of sodium salicylate. Several compounds were analysed for their ability to prevent DNA damage as a consequence of ETP, PHL, and DOX exposure. (**a**) Co-administration of the thiol and antioxidant N-acetylcysteine (NAC) failed to significantly improve the response of ETP-treated MII oocytes. (**b**) Co-incubation of oocytes with sodium salicylate (an inhibitor of topoisomerase IIα) and ETP was capable of mitigating the deleterious impact of ETP treatment at 10 mM. (**c**) Analysis of cytosolic reactive oxygen species did not reveal an appreciable increase following ETP exposure as assessed by the intensity of DCF-DA probe fluorescence. (**d,e**) In addition, co-incubation of sodium salicylate and PHL and DOX proved effective in decreasing the level of DNA damage (γH2A.X fluorescence) generated by these genotoxic compounds. Statistical significance was determined using ANOVA and Tukey–Kramer HSD. Each experiment was conducted on a minimum of three biological replicates, with each replicate comprising a minimum of 30 oocytes. All data are expressed as means ± S.E.M.

The thiol, NAC, has an impressive array of mechanisms by which it can putatively afford protection against DNA damage. These attributes include its capacity to act as a nucleophile (and therefore antioxidant), modulate DNA repair, alter cellular metabolism, influence anti-inflammatory and finally anti-angiogenic activity^42^. Despite these myriad of beneficial actions, supplementation of NAC (2.5 mM^43^) in our culture system failed to significantly improve the response of ETP treated MII oocytes. Thus, γH2A.X related fluorescence remained equivalent in both ETP/NAC exposed oocytes and those exposed to the ETP insult alone (Fig. 8a) (100 μg/ml *P* = 0.2201 and 200 μg/ml *P* = 0.9056). To confirm this result, analysis of the cytosolic ROS response elicited by ETP exposure was undertaken using DCF-DA, a fluorescent probe that reports total cytosolic ROS activity. As illustrated in Fig. 8b, the concentrations of ETP administered herein were unsuccessful in increasing ROS production above that recorded in control populations of oocytes (*P* = 0.935).

The second compound tested was that of SS, the active ingredient in aspirin. As a catalytic inhibitor of topoisomerase IIα, SS has been documented to inhibit genotoxic agent induced topoisomerase IIα-DNA cleavable complex formation *in vivo*^39^. Since ETP also relies on the formation of ETP stabilised topoisomerase IIα-DNA complexes to propagate DNA damage^44^, it was reasoned that pre-treatment of oocytes with SS could mitigate the deleterious impact of ETP treatment. This was precisely the case, with 10 mM SS^39^ proving effective in combating ETP genotoxicity as measured by the intensity of γH2A.X fluorescent foci (Fig. 8c; P = 0.0134). In addition, co administration of SS with PHL (Fig. 8d) and DOX (Fig. 8e) also proved effective at reducing the genotoxicity of these drugs *in vitro* (PHL: P = 0.0076 and DOX: P < 0.0001).

## Discussion

It is widely held that the mammalian oocyte has limited capacity for DNA repair beyond the imposition of cell cycle checkpoints, which serve to arrest development^12,13^. It is therefore curious that these cells are endowed with an impressive suite of proteins that are capable of enacting DNA repair^20,45^. Such findings have promoted the current study in which we have begun to explore the extant molecular repair mechanisms present in the post-ovulatory oocyte as well as the cell’s ability to enact the repair of DSB DNA damage. Our collective data support the presence of an active non-homologous end joining pathway in MII stage mouse oocytes. In addition, we have identified that co-administration of sodium salicylate with the cytotoxic drugs ETP, PHL and DOX, effectively prevents their induction of genomic instability.

Previous reports have established that the oocyte is susceptible to genotoxic assault and the consequential induction of DNA damage^12–16,19,46^. These data agree with the findings of the current study, where we have demonstrated that a range of chemotherapeutic agents, including ETP, PHL and DOX are each capable of eliciting a significant, dose-dependent increase in DNA DSB in MII mouse oocytes. Notably, the impact of such treatments extended beyond DNA damage to include a striking increase in metaphase plate alignment abnormalities and chromatin aggregations. Interestingly however, these irregularities were not accompanied by overt changes in meiotic spindle dimensions. A recent study by Marangos *et al*.^12^ reported phenotypically similar spindle aberrations without an attendant dysregulation of spindle proportions in response to ETP exposure of MI oocytes. In this instance, as many as 80% of the oocytes succumbed to a SAC mediated cell cycle arrest. In view of these data, it is perhaps surprising that the legacy of ETP treatment did not extend to preventing ETP treated MII oocytes from participating in the fertilisation cascade following *in vitro* fertilisation. In this regard, presumptive zygotes appeared morphometrically normal and did not retain γH2A.X labelling despite being generated from a pool of oocytes with significant chromosomal abnormalities.

Downstream of fertilisation, embryonic development assays revealed that ETP did severely compromise early embryonic competency with ~40–60% of embryos failing to progress beyond the two-cell stage of development. Notably, many of these embryos displayed a permanent senescence-like arrest, with little evidence of apoptosis being detected after 5 days of development^47^. This phenomenon mirrors that reported to occur in approximately 10–15% of all human and bovine IVF embryos, which also experience arrested development at the 2- to 4-cell cleavage stages^47^. It has recently been postulated that this permanently-arrested state may constitute part of a quality control mechanism that prevents further development of low-quality embryos^48^. In the context of our study, it is notable that the 2-cell stage coincides with the timing of embryonic genome activation in murine species^49–51^. It is therefore not surprising that embryos harbouring pronounced morphometric abnormalities such as those documented in this study would be removed from the developmental programme at this stage.

In seeking to account for the unequal cytoplasmic partitioning induced by ETP, it is noteworthy that cell division in the mammalian embryo typically remains symmetrical up until at least the 16–32-cell stage^52^. Thus, the formation of large and small blastomeres during earlier developmental phases may be attributed to ETP-mediated dysregulation of the cytoskeletal network responsible for faithful completion of blastomere cytokinesis. This interpretation is consistent with reports that senescence-like phenotypes induced by cytostatic drugs such as ETP (and DOX) are coupled to alterations in the integrity of cytoskeletal elements^28,29^. Despite this, at the level of resolution afforded by confocal microscopy, we did not detect any overt changes in either the labelling intensity or distribution patterns of the actin and tubulin networks assembled within the preimplantation zygote or 2 cell embryos irrespective of the treatment they received. As an alternative explanation, it is known that both the initiation and orientation of the first cleavage is tightly coupled with spindle quality and cleavage symmetry^53^. Indeed, embryos displaying significant spindle abnormalities often initiate incorrect cytokinesis leading to premature developmental arrest^54^. This accords with our findings that the spindle suffered significant misalignment during ETP treatment at the MII stage.

Having established the substantial impact of acute ETP exposure in terms of attenuating the developmental competency of the MII oocyte, we next sought to address whether the MII oocyte is capable of mounting an effective DNA damage repair response to protect the female genome against genotoxic insult. In somatic cell models, DNA repair kinetics are relatively slow, with the interval from initiation to completion generally two to six hours after the primary insult^31^. The loss of γH2AX at DSB sites is widely held to reflect the completion of repair of DNA at break sites^30,55,56^. Accordingly, we tracked the resolution of γH2A.X in mouse MII oocytes over a recovery period of up to 6 hours^30,55^. The kinetic profiling of γH2A.X expression was characterised by a gradual decline to basal levels, equivalent to those of the untreated controls by 4 hours post-treatment. Consistent with data from recent global proteomic analyses of both mouse and human oocytes^20,57^, immunocytochemistry confirmed the presence and localisation of several enzymes that hold key functional roles within the NHEJ pathway, namely; ATM, KU80/XRCC5, DNA PKcs and PCNA. Notably, the expression of the active, phosphorylated forms of both ATM (p-Ser1981) and DNA PKcs (p-Ser2056) were also detected in the MII oocyte. Moreover, the functional significance of NHEJ repair activity was supported by the application of selective pharmacological inhibitors, NU7441 (a selective agonist of DNA-PKcs) and SCR7 (a potent inhibitor of DNA ligase IV); both of which prevented the resolution of γH2A.X foci. These data encourage a reappraisal of the paradigm that, beyond the early follicular phases of development, oocytes are largely refractory to DNA repair^12,13^.

Notwithstanding the potential importance of an active NHEJ in mature oocytes, it was apparent that this reparative pathway did not noticeably rescue the developmental potential of oocytes challenged with an ETP insult. Indeed, embryos generated from ETP-treated oocytes were 6-times more likely to retain significant metaphase plate abnormalities over their untreated counterparts. Moreover, the fact that such irregularities persisted throughout the entire six-hour recovery time suggests that spindle organisation may be refractory to repair and thus contribute to the reduction in developmental capacity of these embryos. An alternative explanation to account for the relative inefficacy of NHEJ in rescuing developmental competence, is that a primary function of the MII oocyte’s DNA surveillance/repair proteins (not restricted to NHEJ) may be the resolution of DNA damage harboured by the fertilising spermatozoon. Indeed, this model has recently been advanced to account for the action of the base excision repair (BER) pathway in mouse MII oocytes^45^.

Irrespective, the finding that ETP-treated oocytes were unable to progress through embryonic development despite harbouring an active NHEJ pathway, prompted us to explore alternative therapeutic interventions to prevent, rather than repair, the associated DNA damage. For this purpose, we assessed the efficacy of co-administration of 2 compounds, N-acetylcysteine (NAC) and sodium salicylate (SS), both of which mitigate the effect chemotherapeutic agents in somatic cell cancers^39–41^, in terms of providing additional protection for the female germline. The thiol NAC has a broad array of activities^42^, including that of an antioxidant; a property that reflects its ability to act as a potent nucleophile. Such activity is of interest in the context of the current study owing to evidence that ETP can elicit a reactive oxygen species (ROS) response following introduction to various tumorigenic cells^58^. However, despite its promise, NAC supplementation failed to significantly reduce the sensitivity of ETP treated MII oocytes to DNA damage; a finding that accords with the failure of ETP to elicit an appreciable ROS response in our experimental regimen. The second compound tested, SS is a catalytic inhibitor of topoisomerase IIα with the potential to block genotoxic agent induced topoisomerase IIα-DNA cleavable complex formation^39^. Since ETP, DOX and PHL have been purported to also rely on the formation of drug stabilised topoisomerase IIα-DNA complexes to elicit DNA damage^44^, it was reasoned that SS could mitigate the deleterious impact of genotoxic insult on the MII oocyte. Indeed, co-administration of SS with ETP, PHL and DOX proved effective in preventing the induction of genomic instability.

## Conclusion

In summary, our data provide the first evidence that the MII oocyte has the potential to conduct DNA repair via NHEJ. Notably, this repair capacity did not prove successful in mitigating the deleterious secondary consequences of genotoxic exposure (i.e. metaphase plate abnormalities), suggesting that there exists a threshold of DNA damage beyond which this pathway becomes insensitive. Nonetheless, our findings provide an intriguing line of inquiry for defining the role of NHEJ as a repair platform for correcting damage of the maternal, and possibly more importantly, the paternal DNA at the moment of fertilisation. Our data also provide the impetus to explore the beneficial effects of co-administration of sodium salicylate, as a means of protecting the female germline during chemotherapeutic interventions.

## Materials and Methods

### Materials

The reagents used during this study were purchased from Sigma Aldrich (St Louis, MO, USA) and all antibodies were from Abcam (Cambridge, UK) unless otherwise stated. Anti-γH2A.X antibodies (SC101696) were obtained from Santa Cruz Biotechnology (Dallas, TX, USA) and anti-XRCC5/KU80 (16389-1-AP) from Proteintech (Rosemont, IL, USA).

### Ethics statement

All research animals in this study were handled, monitored and euthanised in accordance with NSW Animal Research Act 1998, NSW Animal Research Regulation 2010 and the Australian Code for the Care and Use of Animals for Scientific Purposes 8th Ed. as approved by the University of Newcastle Animal Care and Ethics Committee (approval number A-2012-208). C57/BL6/CBA F1 hybrid female mice were bred and held at the institutes’ Central Animal House with food and water ad libitum. Animals were housed under a 12 h light/12 h dark cycle at a constant temperature of 21–22 °C and euthanised immediately before use via CO_2_ asphyxiation.

### Gamete retrieval

Oocytes and spermatozoa were harvested as described previously^19,38^. Briefly, our intraperitoneal injection regimen consisted of the administration of 7.5 IU equine chorionic gonadotropin (eCG) and human chorionic gonadotropin (hCG) (Intervet, Sydney, NSW, Australia) to juvenile (3–5 week old) C57/BL6/CBA F1 female mice to induce superovulation. Thirteen to 15 h after the final injection, female mice were sacrificed via CO_2_ asphyxiation before the immediate retrieval of the cumulus enclosed MII oocytes from oviductal ampullae. Oocytes were subsequently denuded by incubation in 300 µg/ml hyaluronidase at 37 °C for no more than 5 min. Any remaining cumulus cells were removed by a further 3–5 washes in M2 medium.

### Oocyte treatments

To induce DSB DNA damage, MII oocytes were exposed to either 100 or 200 μg/ml concentrations of etoposide (ETP), in parallel with a vehicle (containing the equivalent highest concentration of dimethyl sulfoxide (DMSO)) and an untreated control (M2 media alone) for 15 min at 37 °C as previously described^19^. Alternative treatments included the administration of phleomycin (PLE, 9564) (10 or 50 μg/ml, 1 h), doxorubicin hydrochloride (DOX, PHR1789) (20 or 40 μM, 1 h), N-acetylcysteine (138061) or sodium salicylate (71945), along with their associated vehicle controls supplemented with the equivalent highest concentrations of H_2_O and DMSO, respectively. Following treatment, oocytes were washed in M2 media and either prepared for assessment of DNA damage or for fertilisation using standard *in vitro* fertilisation (IVF) protocols, as described below.

### *In vitro* fertilisation and embryo culture

Following appropriate treatment, denuded MII stage oocytes were liberated from the cumulus matrix and washed in human tubal fluid (HTF) medium 3 times to ensure the complete removal of all ETP reagent (see above) prior to fertilisation. The oocytes were then allocated into a droplet of HTF supplemented with 1 mM reduced glutathione (GSH). Spermatozoa were recovered simultaneously from the cauda epididymides of mature (≥8 weeks) male C57/BL6/CBA F1 mice by retrograde perfusion via the vas deferens and capacitated by incubation in modified Biggers, Whitten and Whittingham (BWW) medium containing 1 mg/ml polyvinyl alcohol and 1 mg/ml methyl-beta cyclodextrin for 1 h at 37 ^°^C under an atmosphere of 5% O_2_, 6% CO_2_ in N_2_ as previously described^38^.

Oocytes were then co-incubated with 2 × 10^5^ capacitated spermatozoa for 4 h at 37 °C. The resultant zygotes or unfertilised oocytes were washed to remove unbound or loosely adherent spermatozoa before being assessed for markers of successful fertilisation (i.e. extrusion of the second polar body and/or pronucleus formation)^19^. In addition, oocytes which arrested at the one cell stage following IVF were assessed via labelling of the nuclear content with DAPI. This strategy enabled us to differentiate between those cells possessing pronuclei (i.e. arrested zygotes) versus those with an intact metaphase plate (i.e. non-fertilised oocytes) and thus increase the accuracy of the fertilisation rate assessment. To promote embryonic development, zygotes were cultured in GSH free HTF medium overnight, before the 2 cell embryos were transferred to G1 PLUS culture medium (Vitrolife, Göteborg, Sweden). After 4 days of culture, embryos underwent an additional transfer into G2 PLUS medium (Vitrolife)^38^. All embryos were monitored on a daily basis and developmental rates recorded. The percentage of oocytes that fertilised and reached the blastocyst stage was calculated on the morning of day 5 post-fertilisation.

### Immunocytochemistry

To ensure access of antibodies to intracellular antigens, oocytes and embryos were first fixed in 3.7% (v/v) paraformaldehyde (1 h) and permeabilised in a solution of 0.25% Triton X-100 diluted in PBS for 10 min at room temperature (RT). Notably, fixation was conducted immediately after the embryos/oocytes had been washed by serial aspiration in fresh M2 medium (i.e. within a maximum of 1–2 min after completion of the appropriate treatment). All cells were then blocked in 3% BSA/PBS for 1 h at 37 °C and incubated in either anti-γH2A.X, anti-ɑ-tubulin (1:400, A11126, Thermo Fisher Scientific, Waltham, MA, USA), anti-ATM (ab82512), anti-p-ATM (phosphor-Ser1981) (ab81292), anti-XRCC5/Ku80, anti-PCNA (ab29), anti-DNA PKcs (ab70250) or anti-p-DNA PKcs (phospho-Ser2056) (ab18192) (each diluted 1:100 in 1% BSA/PBS with the exception of anti-PCNA which was diluted 1:100 in 1% BSA/TBS) antibodies overnight at 4 °C. After primary antibody binding, oocytes and embryos were washed in 1% BSA/PBS prior to incubation with the appropriate Alexa Fluor 488 or 594 conjugated secondary antibodies (Thermo Fisher Scientific) (diluted 1:1000 in 1% BSA/PBS) at 37 °C for 1 h. Dual labelling of oocytes to facilitate morphometric assessment of the meiotic spindle, was achieved by sequential incubation in appropriate primary antibodies prior to the addition of appropriate secondary antibodies in tandem. All cell preparations were counterstained with the nuclear marker, 4′,6-diamindino-2-phenylindole (DAPI) and mounted onto Menzel Glӓser microscope slides (Thermo Fisher Scientific) in antifade reagent (Prolong Gold Antifade, Thermo Fisher Scientific). The intensity of fluorescent labelling was assessed against a secondary antibody only control using microscopy images captured an AXIO Imager.A1 fluorescence microscope (Carl Zeiss Micro Imaging GmbH, Jena, Thuringia, Germany), with all imaging parameters being kept consistent between treatment groups. Briefly, microscopy images were imported into the ImageJ software program and, depending on the nature of the analysis, the ‘circle selection tool’ was used to accurately trace around the entire oocyte or the ‘polygon selection tool’ was used to restrict the analysis to the metaphase plate (defined by the DAPI counterstain); and the mean fluorescence value recorded. As a complementary approach, confocal laser scanning microscopy was utilised to determine the spatial profile of immunofluorescence signals, with all representative images captured using the Olympus FV1000 confocal and a 60×/1.2 NA UPLSAPO oil immersion objective lens (Olympus, Australia). To enable spindle and chromosome configuration analyses, these structures were labelled with a combination of anti-α-tubulin antibody, a fluorescent phalloidin conjugate (which labels polymeric actin), and DAPI (20 min at 37 °C)^59^. The confocal Z stacking function was utilised to record the dimensions of the entire spindle from pole to pole. Finally, the images collected were again imputed into Image J and analysed using a custom spindle analysis tool/macro designed by Dr. S. Lane^60^, thus facilitating calculation of spindle size and length. To avoid operator bias, these analyses were conducted in a blinded manner.

### Embryonic diameter analysis

Confocal microscopy was utilised to capture an optical section through the centre of each blastomere. This image was then imported into Image J in preparation for measurement of cell diameter (as described in the above text). In line with ESHRE recommendations, a threshold of >25% difference in diameter was used to discriminate the percentage of embryos harbouring blastomeres of unequal size^52^.

### DNA repair

To establish whether DNA repair could occur in MII oocytes, cells were treated with ETP, washed and then provided with a 6 h recovery period in fresh M2 media. A subset of cells were removed at each time point and assessed for the extent of DSB DNA damage (γH2A.X). To provide functional evidence of DNA repair occurring via non-homologous end-joining (NHEJ), a separate subset of oocytes were subjected to co-incubation with both ETP and an agonist of NHEJ for the duration of the recovery period. In these experiments, agonists consisted of either NU7441/KU57788 (50 μM; S2638, Selleckchem, Houston, TX, USA) a potent and selective inhibitor of DNA-PKcs or SCR7 (20 μM; M60082, Xcessbio Biosciences, San Diego, CA, USA) a specific inhibitor of DNA ligase IV. Thereafter, oocytes were fixed and examined for indicators of DNA damage and metaphase plate misalignment (as previously described).

### SDS-PAGE and immunoblotting

Ultra violet light treated HELA cell (ab157396) and HEK293 (ab7902) protein lysates were sourced from commercial suppliers and diluted to a working concentration of either 10 μg/ml (anti-XRCC5/Ku80, anti-PCNA and anti-ATM) or 20 μg/ml (anti-ATM, anti-DNA PKcs and anti-p-DNA PKcs (phospho-Ser2056)) with SDS-PAGE loading buffer. The protein lysates were then resolved on pre-cast polyacrylamide gels (4–12% NuPAGE Bis-Tris, Thermo Fisher Scientific), prior to transfer to either nitrocellulose or polyvinylidene difluoride (PVDF: anti-XRCC5/Ku80) membranes. Membranes were blocked with 5% LFDM (low fat dried milk) or 5% BSA (anti-ATM) for 1 h, washed in Tris-buffered saline (TBS) containing 0.1% Tween (TBST) and then sequentially incubated in the appropriate primary antibody (diluted 1:1000 in 1% BSA or LFDM in TBST) overnight at 4 °C and HRP-conjugated secondary antibodies (diluted 1:1000 in 1% BSA or LFDM or TBST) for 1 h at room temperature^61^. Membranes were developed using enhanced chemiluminescence reagents (GE Healthcare, Buckinghamshire, England UK) according to the manufacturer’s instructions.

### Cytosolic reactive oxygen species (ROS) measurement

Assessment of cytosolic ROS levels were conducted following ETP administration using the 5′-carboxy-2′,7′-difluorodihydrofluorescein diacetate (DCF-DA probe) (Molecular Probes, OR, USA) as previously described^37,62^. Briefly, control and ETP treated MII stage oocytes were incubated in a 10 μM solution of DCF-DA in M2 media for 30 min at 37 °C. Oocytes were then washed 5 times before mounting and analysis by fluorescence microscopy^37,62^.

### Statistical Analysis

Densitometric analysis and quantification of fluorescence levels within oocytes and pronuclear stage zygotes was achieved using the public-sector image processing program, ImageJ (National Institute of Health, Maryland, USA). Statistical significance was determined using ANOVA, Tukey–Kramer HSD and Student’s t-tests employing JMP (version 13.0.0, SAS Institute, NC, USA) and Excel software (Version 15.32, Microsoft, Washington, US). Differences with a value of *P* < 0.05 were considered to be statistically significant. Each experiment was conducted on a minimum of three biological replicates. All data are expressed as mean ± S.E.M.

### Data Availability

All data generated or analysed during this study are included in this published article (and its Supplementary Information files).

## Electronic supplementary material

Supplementary Figures
