# Supplementary material for: Double Strand Break DNA Repair occurs via Non-Homologous End-Joining in Mouse MII Oocytes
Source: Sci Rep. 2018 Jun 26;8:9685. doi: 10.1038/s41598-018-27892-2 (PMC6018751; doi:10.1038/s41598-018-27892-2)

**TITLE: Double strand break DNA Repair occurs via Non-Homologous End-Joining in Mouse MII Oocytes**

**AUTHORS:** Jacinta H Martin^1,2*^, Elizabeth G Bromfield^1,2^, R. John Aitken^1,2^, Tessa Lord^3^, and Brett Nixon^1,2^

**AUTHOR AFFLIATIONS:** ^1^Priority Research Centre for Reproductive Science, School of Environmental and Life Sciences, the University of Newcastle, Callaghan, NSW 2308, Australia. ^2^Preganancy and Reproduction Program, Hunter Medical Research Institute, New Lambton Heights, NSW, Australia, 2305. ^3^School of Molecular Biosciences, Centre for Reproductive Biology, College of Veterinary Medicine, Washington State University, Pullman, WA, USA, 99164.

^*^**CORRESPONDENCE**:

Jacinta H Martin

Email: Jacinta.martin@newcastle.edu.au

Phone: +61 2 4921 2043

Fax: +61 2 4921 6308


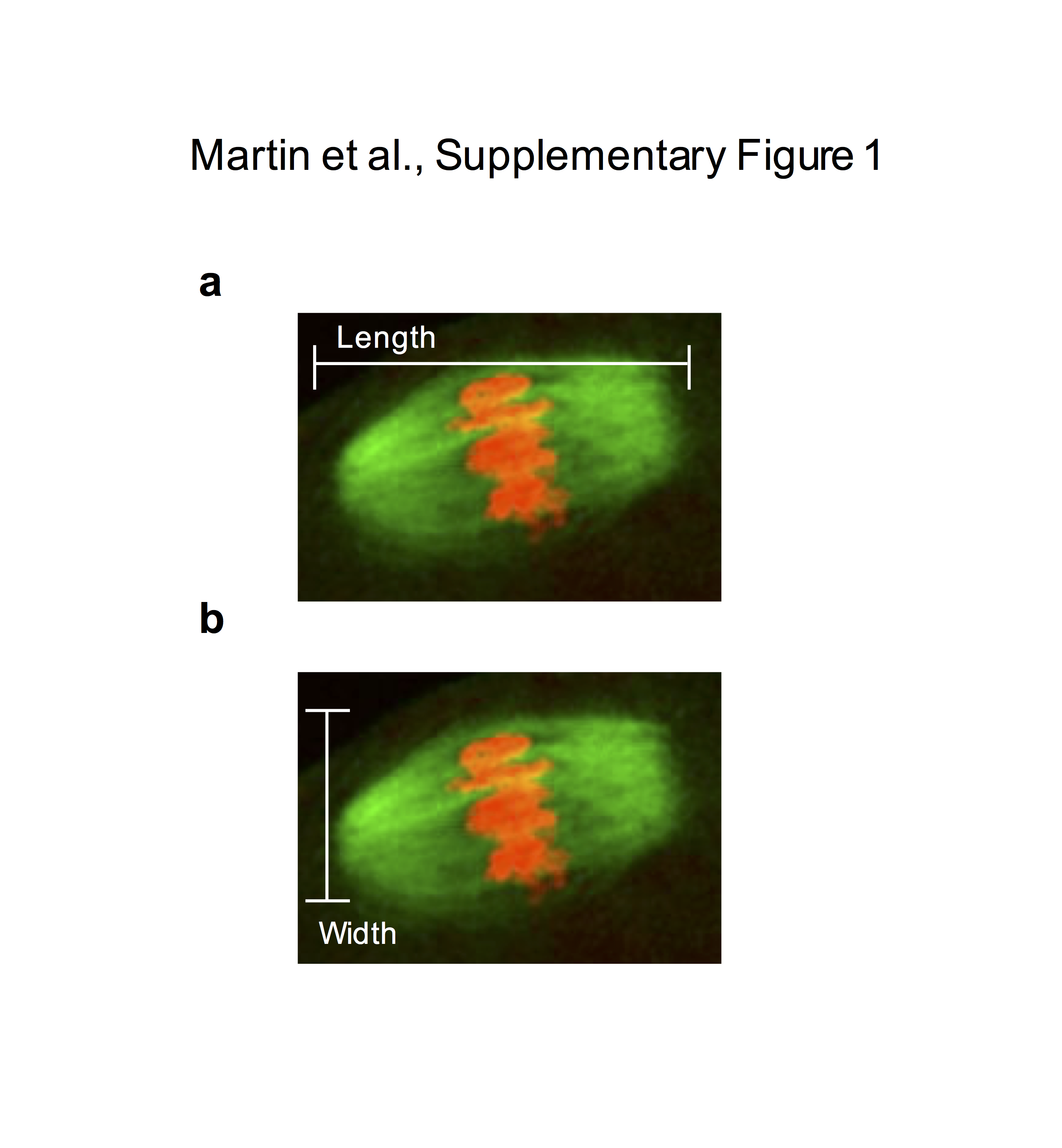


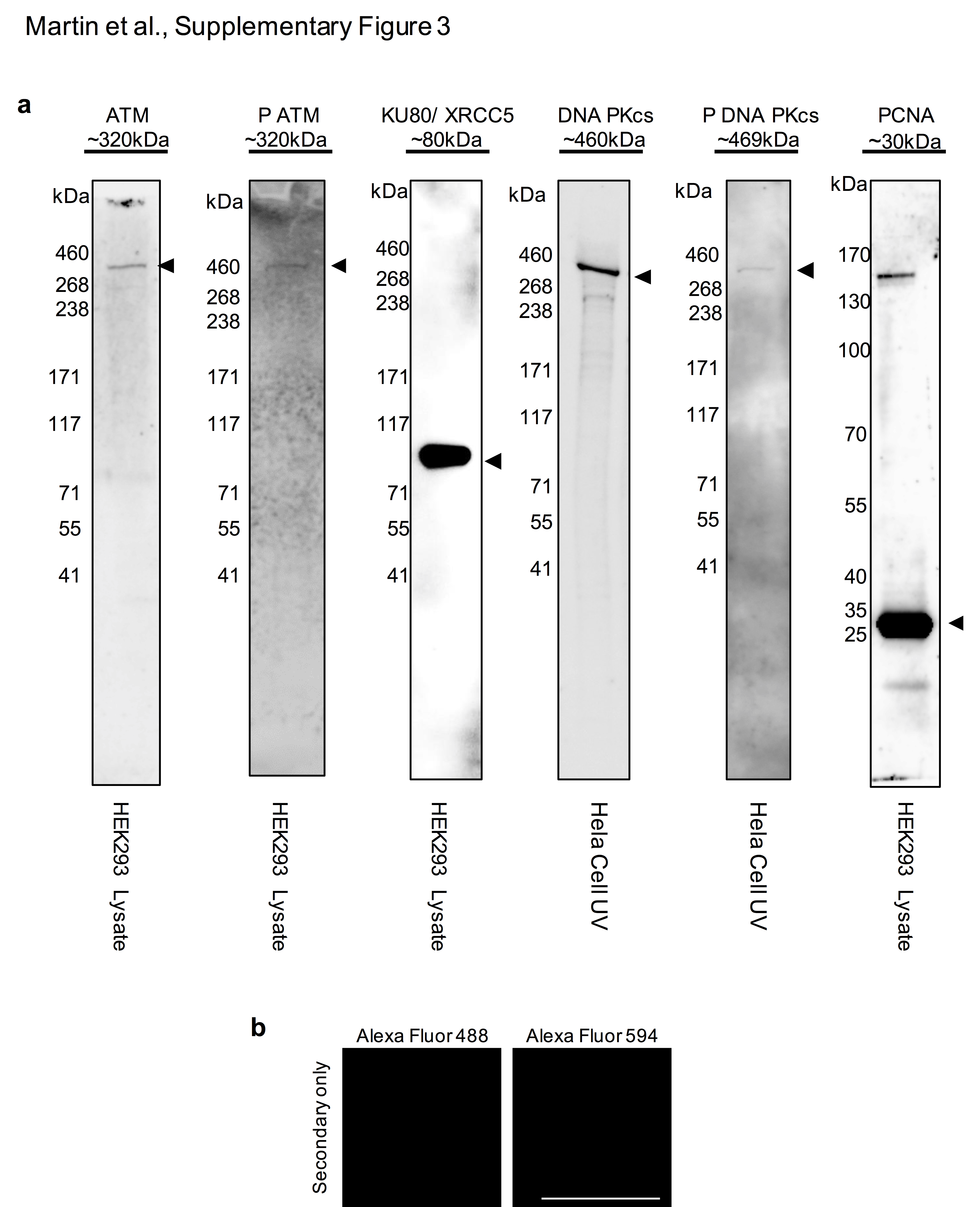


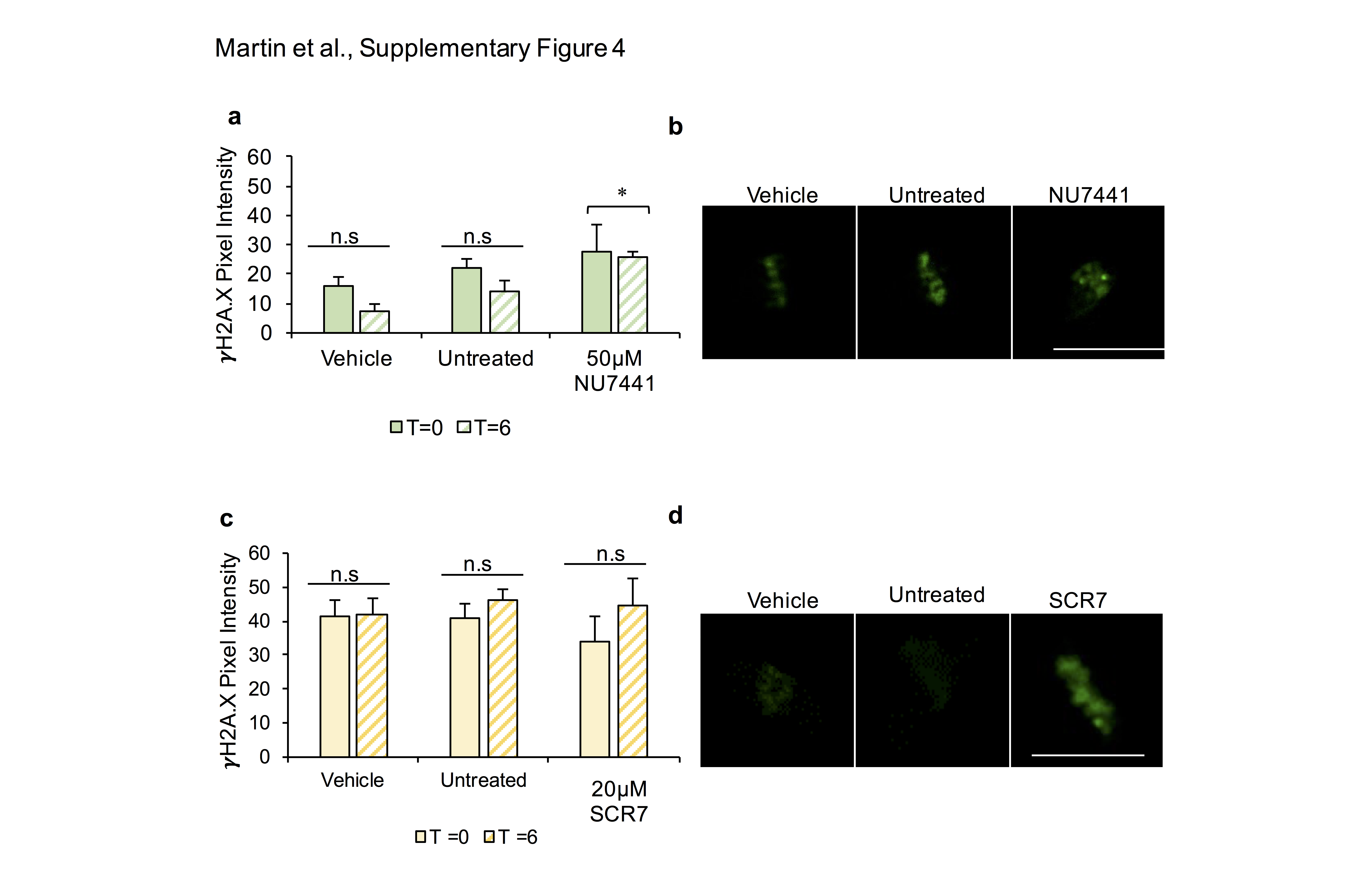

Supplement: Supplementary file 1 — Supplementary Figures [file 41598_2018_27892_MOESM1_ESM.docx]
